# Importance and effectiveness of correction methods for spatial sampling bias in species with sex‐specific habitat preference

**DOI:** 10.1002/ece3.5765

**Published:** 2019-11-19

**Authors:** Anouk Glad, Jean‐Matthieu Monnet, Jörn Pagel, Björn Reineking

**Affiliations:** ^1^ Univ. Grenoble Alpes Irstea LESSEM Grenoble France; ^2^ Groupe Tétras Jura Les Bouchoux France; ^3^ Institute of Landscape and Plant Ecology University of Hohenheim Stuttgart Germany

**Keywords:** correction methods, sex ratio, spatial sampling bias, species distribution model

## Abstract

**Aim:**

Presence records from surveys with spatially heterogeneous sampling intensity are a key challenge for species distribution models (SDMs). When sex groups differ in their habitat association, the correction of the spatial bias becomes important for preventing model predictions that are biased toward one sex. The objectives of this study were to investigate the effectiveness of existing correction methods for spatial sampling bias for SDMs when male and female have different habitat preferences.

**Location:**

Jura massif, France.

**Methods:**

We used a spatially sex‐segregated virtual species to understand the effect of three sampling designs (spatially biased, uniform random, and systematic), and two correction methods (targeted background points, and distance to trajectories) on estimated habitat preferences, sex ratios, and prediction accuracy. We then evaluated these effects for two empirical Capercaillie (*Tetrao urogallus*) presence‐only datasets from a systematic and a spatially biased sampling design.

**Results:**

Sampling design strongly affected parameter estimation accuracy for the virtual species: noncorrected spatially biased sampling resulted in biased estimates of habitat association and sex ratios. Both established methods of bias correction were successful in the case of virtual species, with the targeted correction methods showing stronger correction, as it more closely followed the simulated decay of detectability with distance from sampling locations. On the Capercaillie dataset, only the targeted background points method resulted in the same sex ratio estimate for the spatially biased sampling design as for the spatially unbiased sampling.

**Main conclusions:**

We suggest that information on subgroups with distinct habitat associations should be included in SDMs analyses when possible. We conclude that current methods for correcting spatially biased sampling can improve estimates of both habitat association and subgroup ratios (e.g., sex and age), but that their efficiency depends on their ability to well represent the spatial observation bias.

## Graphical abstract

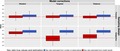

## 1 INTRODUCTION

The impact of human activities on the environment at different scales, from local (urbanization and agriculture) to global (climate change), necessitates to better understand the relationships between species and their environment, and to better predict their distribution. Species distribution models (SDMs) are an important tool in ecology and conservation biology (Franklin, 2009a, 2009b; Johnson & Gillingham, 2005), as they link organism occurrences to spatial environment characteristics (Elith & Leathwick, 2009). Additionally, SDMs can predict spatial distributions that are used to plan conservation actions, wildlife management, and monitoring strategies (e.g., new sampling designs).

Occurrence data for distribution modeling come from many sources and often do not originate from controlled sampling survey designs such as systematic transects or random plots, but from spatially preferential sampling or opportunistic observations (Geldmann et al., 2016). Data collected without a sampling framework are subject to sampling bias (Guisan, Thuiller, & Zimmermann, 2017), which can be of different sources: uneven record intensity in space or time and uneven sampling effort and variations in detection efficiency among observers (Geldmann et al., 2016). In this study, we focus on the effects of uneven record intensity in space, since spatially biased sampling is a major cause of poor model predictions accuracy (Araújo & Guisan, 2006; Guisan & Zimmermann, 2000; Renner et al., 2015). Spatial sampling bias often occurs in the absence of a predefined sampling scheme, because observers tend to survey areas depending on their personal preferences, influenced for example by accessibility, higher potential of observations, or previous knowledge of the study area (Isaac & Pocock, 2015). Since the characteristics of sampling bias are specific to each dataset and their influence is unknown most of the time, it must be approximated in the statistical analysis. Consequently, the effect of sampling bias on prediction may often be underestimated, as many published results are not corrected for sampling bias before analysis (Yackulic et al., 2013).

Multiple methods have already been developed to correct sampling bias and improve models and predictions. Phillips et al. (2009) proposed the targeted background method which distributes background points in space with the same bias as the observer process. It has been used in numerous studies (Kramer‐Schadt et al., 2013), and it was shown to give better results than randomly distributed background points (Warton, Renner, & Ramp, 2013). However, when the targeted background points are generated over a too restricted area, it can also reduce model accuracy and results in lower prediction performances (Fourcade, Engler, Rödder, & Secondi, 2014; Thuiller, Brotons, Araújo, & Lavorel, 2004; Warton et al., 2013). As an alternative, Cardador, Diaz‐luque, Hiraldo, Gilardy, and Tella (2017) recently proposed to include the potential causes of bias as predictor variables in the models, in combination with a random background sampling.

Models created from spatially unbiased datasets and models successfully corrected for spatial sampling bias are thought to represent the species distribution over the study area, with the underlying hypothesis that all individuals are sampled with the same probability, thus covering the panel of different habitat needs within the population. This assumption can be wrong in the case of spatially biased sampling that involves spatial segregation within species subgroups (e.g., sex and age). Those differences in habitat selection are well‐known for a variety of species. For example, multiple studies showed that differences in habitat preference between sexes exist in avian species and depend on multiple parameters like dispersal capacities, food needs, mating system, and morphology (Cody, 1985). Conde et al. (2010) investigated the case of the jaguar in Central America and concluded that the use of a nonsexed model underestimated the effect of fragmentation on female habitat use, which is an important parameter when planning conservation actions. Different seasonal habitat uses were also observed for female and male bats (Hayes, Cryan, & Wunder, 2015). A first consequence of ignoring such differences in habitat selection between sexes may be that bias correction fails, resulting in incorrect estimates of habitat preferences and of spatial species distribution. A second consequence may be that sex ratios are wrongly estimated, when sampling was preferentially conducted in habitat that is selected by only one sex. The relative abundance of different subgroups, notably the sex ratio, can provide important information on the ecology and conservation status of a species (van Toor, Jaberg, & Safi, 2011). Therefore, taking into account sex‐specific habitat preferences, when correcting for spatial sampling bias, may provide a double benefit of improved estimates of habitat association and species distribution on the one hand and improved estimates of sex ratio (or other subgroup ratios) on the other. Despite these potential benefits, the effect of spatial sampling bias on the reliability of predictions when working on spatially segregated sexes has been little studied.

A known example for different habitat preferences of males and females is the Capercaillie (*Tetrao urogallus*). The species is listed as least concern worldwide, but a population constituted of approximately 300 individuals in the French Jura massif is critically endangered (http://www.observatoire-galliformes-montagne.com/Grand-Tetras.html). Given their size, weight, and color, the male's strategy to avoid predation is known as “detect predator and escape” or “detect predator and self‐defense,” whereas females prefer to hide in dense vegetation of boreal forests (Gjerde & Wegge, 1989; Rolstad, 1988; Rolstad, Wegge, & Larsen, 1988). The same tendency where males use more open areas than females was observed in the Jura massif and in the Alps (Storch, 1993; Thiel, Unger, Kéry, & Jenni, 2007). Management guidelines tend to highlight only the male‐preferred habitats (M. Montadert, personal communication). In addition, the fact that presence signs are easier to find in more open areas, together with an underestimation of the differences in habitat use between sexes, may have led observers to intentionally concentrate the sampling effort on those habitats preferred by males. This is exemplified by a study on Capercaillie genetics by Mollet, Kéry, Gardner, Pasinelli, and Royle (2015), where observers were encouraged to focus on roosting and feeding trees, on hiding sites, on internal forest edges, and on root plates and on tree stumps. Indeed, assuming that the true sex ratio is 0.5, in that study the estimated sex ratio was biased toward males, reflecting the low proportion of female signs found by this sampling protocol. A second study found the same results (Morán‐Luis et al., 2014) for the Cantabrian Capercaillie (*Tetrao urogallus cantabricus*) subspecies.

In this study, we evaluated the effect of two correction methods (targeted background points and distance to trajectories variable) in the case of uneven spatial sampling on estimates of habitat preferences, sex ratio estimation, and prediction accuracy. We compared the correction effect using different survey designs (subjective and systematic) and model types (sex specific and generic) in the case of sex spatially segregated subgroups using both a virtual species and the case study of Capercaillie, where sexes are reported to have preferences for slightly different habitat type (Catry, Campos, Almada, & Cresswell, 2004; Ruckstuhl & Neuhaus, 2000; Wolf, Kauermann, & Trillmich, 2005).

We hypothesized that using spatially biased sampling leads to biased estimates of habitat association (H1) and to a biased sex ratio within the virtual population (H2), but that these biases can be corrected by the two methods introduced above (H3) and that sex‐specific models will give more accurate predictions than standard, generic models (H4). In addition, from our knowledge of previous studies on Capercaillie habitat use, we hypothesized that in the absence of a systematic survey protocol the datasets will be biased toward males (H5), but that sampling bias correction methods can correct the sex ratio estimation (H6). We also hypothesized that sex‐specific models will give more accurate predictions than generic models which will be biased toward males (H7). The general hypotheses H1–H4 were assessed using a virtual species approach, whereas Capercaillie‐specific hypotheses H5–H7 were tested with empirical data.

## 2 METHODS

### 2.1 Study areas

**Figure 1:**
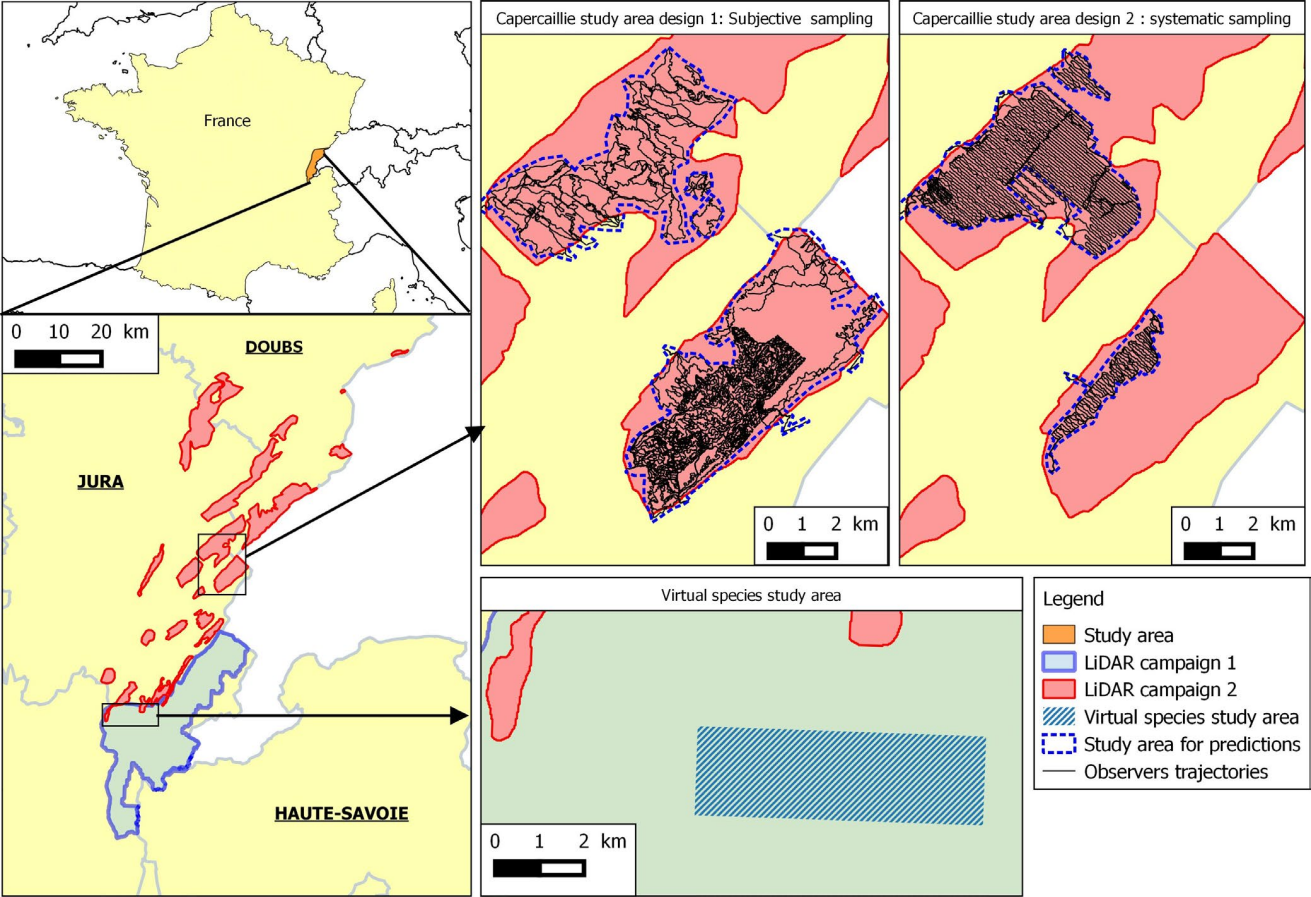
Study areas with LiDAR surveys, Capercaillie sampling designs, and localizations for virtual species case study

The study areas are located in the French Jura massif, within the departments of Ain, Doubs, and Jura (Figure 1). The landscape is composed of a mosaic of small urban areas, pastures, forests, and fields. The massif is composed of a low plateau (elevation range from 400 m to 700 m a.s.l.) and a high plateau (elevation range from 700 m to 1,620 m a.s.l.). For the virtual species, the study area is located in the Ain department, within a 13 km^2^ area in the forest of Champfromier. In the case of Capercaillie, the study area is located in two forests (Risoux and Mont Noir) that were surveyed using both subjective and systematic surveys.

### 2.2 Environmental variables

The environmental variables used in the study for both the virtual species and the Capercaillie models were derived from LiDAR datasets stemming from two acquisition campaigns over distinct departments (Figure 1). The first LiDAR campaign was conducted in fall 2014 and covered 626 km^2^ of the Ain department (France). The second LiDAR campaign was conducted in summer 2016 and covered a surface area of 431 km^2^ in two other French departments, Jura and Doubs. LiDAR point‐cloud metrics were calculated with R package lidaRtRee (https://gitlab.irstea.fr/jean-matthieu.monnet/lidaRtRee). First, LiDAR raw point clouds (classified in ground and vegetation) were normalized with the Delaunay interpolation method from the lidR package (Roussel & Auty, 2016). Point‐cloud summary metrics were calculated as environmental variables within pixels of size 25 m × 25 m.

In the virtual case study, three environmental variables were selected to create the distributions: the Simpson index of vegetation heights, the canopy density between 10 and 20 m height, and slope. Together, these variables capture different aspects of the landscape (vegetation height heterogeneity, vegetation density, and topography) and were chosen because all three variables vary widely across the study area. The correlation between the variables was low: Pearson's correlation coefficients were 0.18 (Slope/Simpson), 0.12 (Simpson/Canopy density), and 0.21 (Slope/Canopy density). In the case of Capercaillie, six variables were selected to capture both vertical and horizontal heterogeneity of the vegetation structure (Graf, Mathys, & Bollmann, 2009). In order to correspond best with the species' home range requirements (Storch, 1997), the mean and the standard deviation of the selected variables were calculated at the scale of 1.8 ha using a circular moving window centered on each pixel (25 m × 25 m). The final selected variables were the mean canopy density between 10 m and 20 m height, the mean penetration ratio between 2 m and 5 m height, the mean Simpson index for height, the standard deviation of the canopy density between 20 m and 30 m height, and the standard deviation of the penetration ratio between 2 m and 5 m height.

### 2.3 Virtual species case study

A virtual species showing sexual differences in habitat preference was simulated by selecting different parameter values for each sex. Hundred replicated simulations of presence for each sex were created, and virtual datasets were collected according to three sampling schemes. Then, SDMs were parameterized using three different methods.

#### 2.3.1 Species distribution intensity

Virtual species presence‐only datasets were created following an inhomogeneous Poisson point process model using the three environmental variables for each sex separately. All variables were normalized by subtracting mean pixel value and dividing by the standard deviation before modeling. The intensity *λ*(*s*) represents the expected number of individuals per spatial unit (pixel) at position s, and it is computed as:

$$\ln\lambda \left(s\right)=\beta_{0}+\sum_{i=1}^{p}x_{i}\left(s\right)\times \beta_{i}$$

where *x_i_*(*s*) represents the environmental covariate *i* at location *s*, and the *β_i_* are the corresponding model coefficients for the effects of the three environmental variables (slope, canopy density, and Simpson index, i.e., *p* = 3), and *β*_0_ denotes the intercept term (Renner et al., 2015). The coefficients *β*_0_ and *β_i_* were chosen in order to obtain intensity values per pixel between 0 and 2 (Table 1). From the intensity map, the number of individual presences per pixel was independently drawn from a Poisson distribution with mean *λ*(*s*).

**Table 1:** Parameter values for the virtual species female and male

	Slope	Canopy density 10–20 m	Simpson index	Intercept
Female	−0.9	−0.8	1	−4
Male	−0.2	0.9	−0.9	−3.55

As we study the influence of different habitat associations between sexes, we did not create coefficients for the distribution of the entire population. Occurrence distributions of both sexes were generated with different *β_i_* coefficients. The intercept *β*_0_ was set for male in order to have a known sex ratio $\left(\text{Nobs}\;\;\text{females}/\text{Nobs}\;\text{females}+\text{Nobs}\;\;\text{males}\right)$ of 0.5 in the study area (Table 1).

#### 2.3.2 Virtual sampling

**Figure 2:**
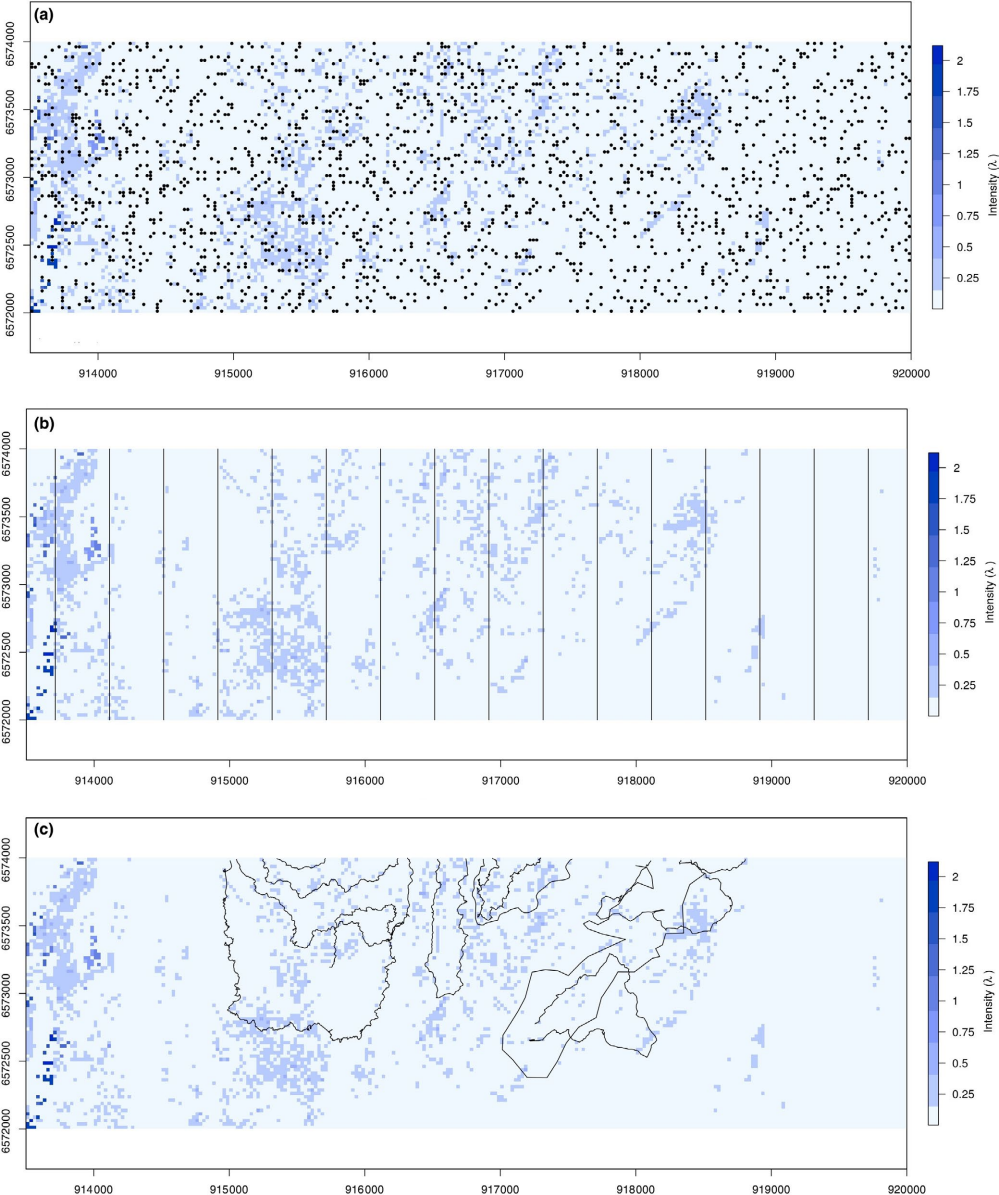
Overview of the three sampling designs for the virtual species case study over the intensity distribution map: (a) random, (b) transects, and (c) subjective

In order to obtain different virtual datasets, three basic sampling designs (random, systematic, and subjective) were applied for each sex (Figure 2). The random sampling was done using homogenous sampling of 2,000 random points within the entire area. The systematic sampling was created using parallel transects separated by 400 m generated with the R package DSsim for a total length of 32 km. For the subjective sampling design, trajectories of real Capercaillie surveys over the selected area were used (total length 31.2 km). The comparison of the density curves among the sampling designs showed a spatial sampling bias in the subjective design, where some environmental values were over‐ or under‐represented relative to their availability in the study area (Figure A1.1).

In a first step, the number of observed occurrences with the different sampling schemes was calculated. For all cases with known trajectories (i.e., the two nonrandom designs), the observation dataset was created from the realized presences (or signs) distribution by adding a distance‐dependent detection probability. The detection probability was maximal on the tracks and diminished with increasing distance from the tracks. The detection probability followed a logspline distribution fitted to empirical distances of sign locations to the observer trajectory. In a second step, for all cases, a random binomial draw was applied, with the virtual species count taken as the number of trials per pixel and distance‐dependent detection probability as probability of success. The resulting count data were converted into presence‐only records by removing the locations with no observations. Locations with more than one observation were replicated according to the number of signs found at the location as in the simulated dataset, the different observations correspond to different individuals occurring in the same pixel. The mean numbers of observations for males and females, respectively, were 108; 105 (random design), 67; 74 (systematic design), and 46; 98 (subjective design).

### 2.4 Capercaillie case study

#### 2.4.1 Datasets description

##### Design 1: Subjective sampling

Long‐term Capercaillie winter surveys were organized without a strongly prescriptive framework between 2007 and 2015 by the “Groupe Tétras Jura” nongovernmental organization. Observers surveyed forests known as favorable for Capercaillie by navigating through the focus area according to observers' preferences. Using recreational‐grade global navigation satellite system (GNSS) receivers, they recorded their survey trajectories and Capercaillie sign locations (mostly feces, prints, and feathers). Whenever possible, sex was assigned to each observation. This can be done with a fairly high accuracy due to the species' high sexual dimorphism; according to preliminary results from a genetic analysis, signs such as droppings can be visually assigned to sexes with a 90% accuracy (A. Depraz, personal communication). A total of 29 different days were dedicated to the collection of the data with a dozen of different observers covering 843 km and a surface area of 4,864.5 ha. All unassigned observations were removed (21 observations), and the 318 remaining observations were used for the analysis (211 males and 107 females).

#### 2.4.2 Design 2: Systematic sampling

The implementation of a new survey protocol in the winters of 2016 and 2017 for a large scale Capercaillie genetic survey involved the collection of droppings following a standardized predefined path. Transect trajectories were separated by 80 m (e.g., coordinates of a given transect 930115,6614707; 931728,6613351 EPSG:2154), and observers were requested to stay within 20 m of their assigned transect. As in design 1, real observer trajectories as well as sign locations were recorded using a GNSS receiver. A total of 4 days were dedicated to the collection of the data with three different observers, covering 412.3 km and a surface area of 2,280.5 ha. A total of 29 observations where the sex was determined were collected (17 males and 12 females and one unassigned).

### 2.5 Modeling

Maxent is arguably the most frequently used SDM method designed to handle presence‐only data (Elith et al., 2006, 2011; Phillips, Anderson, & Schapire, 2006). Here, we use a recent reimplementation of the algorithm building on the equivalence of Maxent with an infinitely weighted logistic regression (Maxnet R package; Phillips, 2017). All analyses were conducted using R 3.4 (R Core Team, 2016). For both the virtual species and the Capercaillie dataset, models were created using female and male observations separately to create sex‐specific models. In addition, generic models combining both female and male observations were created for each case. The performance of each model was assessed by the area under curve (AUC) using the presence data combined with a unique targeted background dataset, different from the one used in the models with targeted background sampling, but following the same distance distribution. The rationale is that this provides the least biased representation of presence records relative to the overall landscape given the observation process. The variance of AUC estimates was calculated using a 10‐fold cross‐validation (LeDell, Petersen, & Laan, 2015).

### 2.6 Bias correction methods

Two different bias corrections methods were applied to both the systematic and the subjective sampling designs: targeted background points and a distance to trajectories variable. For the targeted background point method, 10,000 background points were randomly sampled along transects, where the frequency of distances between each background point and transect followed the same logspline distribution as the one used to generate the detection probability. The second bias correction method was applied by using the distance to trajectories as a predictor variable in the model, combined with 10,000 uniformly random distributed background points. Both correction methods were compared with a noncorrected model with uniformly random background points. The locations of the background points were the same across model replicates.

### 2.7 Parameter estimation (virtual species only)

The aim of this section is to estimate the bias in habitat association (H1) and the effect of sampling bias correction (H3). To control the effect of the bias correction methods on model accuracy, the estimations ${\hat{\beta }}_{i}$ from the fitted models were compared with the true values *β_i_* used to generate the intensity map for each virtual sex. Parameters were estimated for both virtual species sexes, for all three environmental variables. Only the results of females are presented here (for males see Figure A2.1).

### 2.8 Species sex ratio estimation

The aim here is to estimate the sex ratio bias (H2) and the effect of corrections (H3). In the case of the virtual species, the species sex ratio was calculated with two approaches: first, from the number of observations sampled from the virtual sampling, *N*_obs_, and second from the expected total number of signs in the study area that would be observed under complete sampling, *N*_tot_. This expected total number of reported signs was calculated using the predicted relative abundances per cell, *μ_j_*, the number of observations *N*_obs_, and the probability of detection per cell, *p*_obs,j_.

The total number of sample signs *N*_obs_ is the sum of observed signs per cell over all S sampling units (cells):

$$N_{\text{tot}}=\frac{N_{\text{obs}}}{\sum_{j=1}^{s}\mu_{j}p_{\text{obs},j}}$$

where the sum is taken over the *S* cells in the landscape.

The relative abundances per cell, *μ_j_*, sum up to one. The values of relative abundances per cell are obtained from the predictions of the Maxent model for the whole study area, using the “raw” output, and dividing by the sum of predicted raw values. For the model with distance to trajectories correction, the distance variable is set to zero for the whole landscape.

The probability of detection, *p*_obs,_ *_j_*, for a given cell is calculated using the distance to trajectories of that cell, *d_j_*, and using either the logspline density at that distance (for the no correction models and the targeted background method) or calculating the detection probability as the raw output from the distance to trajectories model by setting all nondistance‐related variables to zero. In our particular case, we simply used distance as a linear predictor, and *p*_obs,_ *_j_* can thus be calculated from the distance to trajectories, *d_j_*, and the parameter estimate for the distance to trajectories, *β*_d_, as *p*_obs,_ *_j_* = exp(*β*_d_ *d_j_*). In the case of random sampling design, the *p*_obs,_ *_j_* are taken as 1 in all sampled pixels.

Given the total number of expected observations in the landscape, *N*_tot_, the relative abundance of signs per cell, *μ_j_*, and the size of cells, *a_j_*, we can calculate the density of signs in a cell, *λ_j_*, expressed in number of signs per ha, as

$$\lambda_{j}=\mu_{j}\frac{N_{\text{tot}}}{a_{j}}.$$

In the case of Capercaillie, the sex ratio was estimated from expected total number of signs under complete sampling *N*_tot_, using the same method as presented for the virtual species (hypotheses H5 and H6).

### 2.9 Predicted maps

The relative intensity predicted maps from both sex‐specific models, and generic models were compared with the simulated distribution using the Spearman correlation coefficient (hypothesis H4 and H7). In the case of Capercaillie, the expected number of signs per ha was predicted for each pixel. Predictions from sex‐specific models were compared to the generic model.

## 3 RESULTS

### 3.1 Virtual species case study

#### 3.1.1 Model performance

Overall model AUC ranged between 0.54 and 0.82 (Table A3.1). Female models performed better than male and generic models. For both female and male models, the lowest performances were observed for the subjective design (AUC < 0.58). Generic models showed poor performances with the random and systematic sampling design with and without corrections (AUC < 0.56).

#### 3.1.2 Parameter estimation

**Figure 3:**
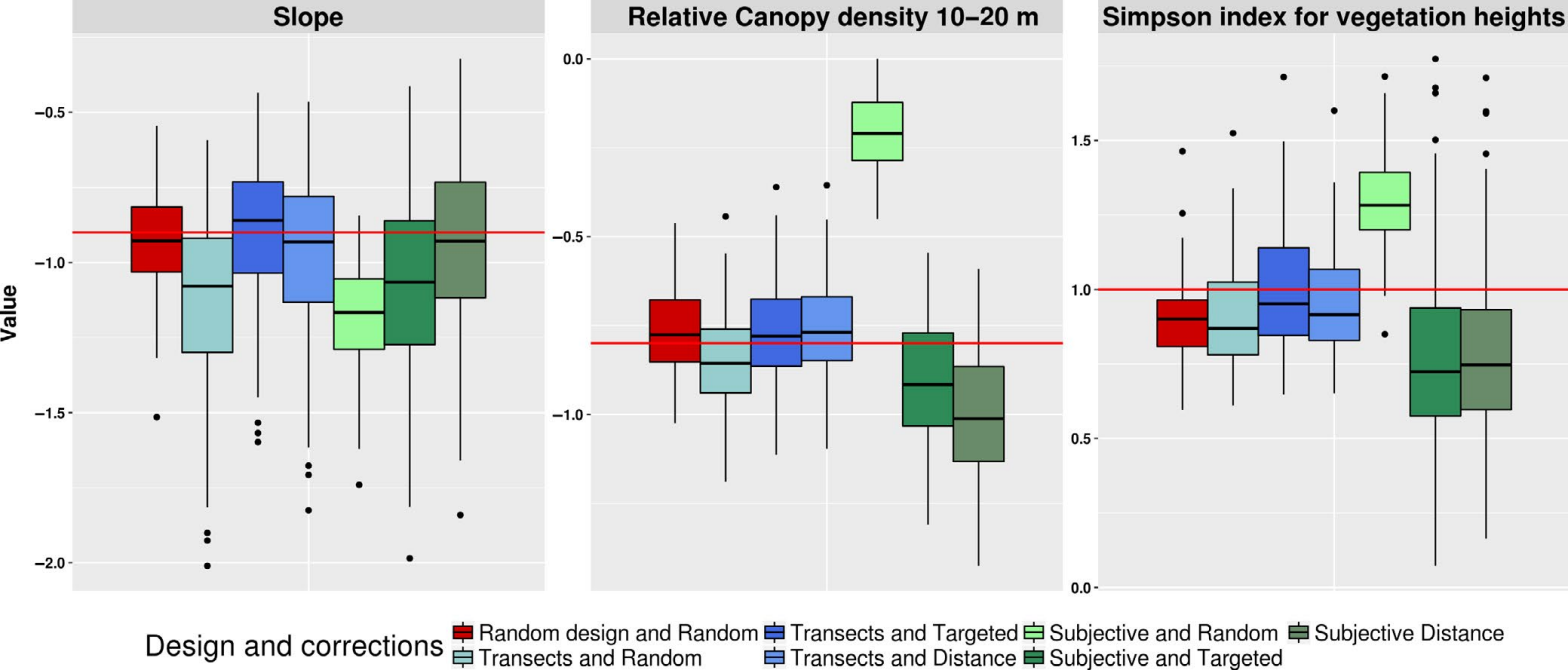
Parameter estimations (from 100 replicate models) for virtual species female. The red line represents the true parameter value

Mean parameter estimates of noncorrected models and corrected models were compared with the true parameter value (Figure 3). The estimated parameters differed significantly from the true value in most cases and there was considerable variation in the magnitude of those discrepancies across scenarios. Parameter estimation for the random design and random background points showed a mean of estimated parameters close to the true parameter values (differences mean_slope = 0.03; mean_canopy = 0.04, mean_simpson = −0.1), for the three variables in the female case. When using the transect design without correction, the estimates differed more strongly from the true values in particular for the variable slope (differences mean_slope = −0.24, mean_canopy = 0.05, mean_simpson = −0.08). With targeted background point correction, the estimated values of the three variables were closer to the real value (differences mean_slope = 0; mean_canopy = 0.03, mean_simpson = 0; Table 2). In the case of the distance correction methods, the parameters were better estimated than the random design for the simpson variable (differences mean_slope = −0.09, mean_canopy = 0.04; mean_simpson = −0.04). Nevertheless, there were minor variations between the three correction cases with systematic designs, and estimates were still relatively close to the true parameter value (absolute difference < 0.04). For the subjective design, without corrections (random background points), the deviations of estimated parameters from the true value were much larger (differences mean_slope = −0.28, mean_canopy = 0.59, mean_simpson = 0.3). This discrepancy (<0.6) was reduced for the three variables by the two correction methods for each variable (differences targeted: mean_slope = −0.27, mean_canopy = −0.1, mean_simpson = −0.18; difference distance: mean_slope = −0.03, mean_canopy = −0.19, mean_simpson = −0.19). Overall, the variance increased when estimating the parameters with sampling bias correction.

**Table 2:** Parameter estimation mean values for female

Design	Correction	Parameter estimates		
		Slope	Canopy	Simpson
Random	Random	−0.93	−0.76	0.90
Systematic	Random	−1.14	−0.85	0.92
	Targeted	−0.90	−0.77	1
	Distance	−0.99	−0.76	0.96
Subjective	Random	−1.18	−0.21	1.3
	Targeted	−1.07	−0.90	0.82
	Distance	−0.93	−0.99	0.81

#### 3.1.3 Sex ratio estimation

**Figure 4:**
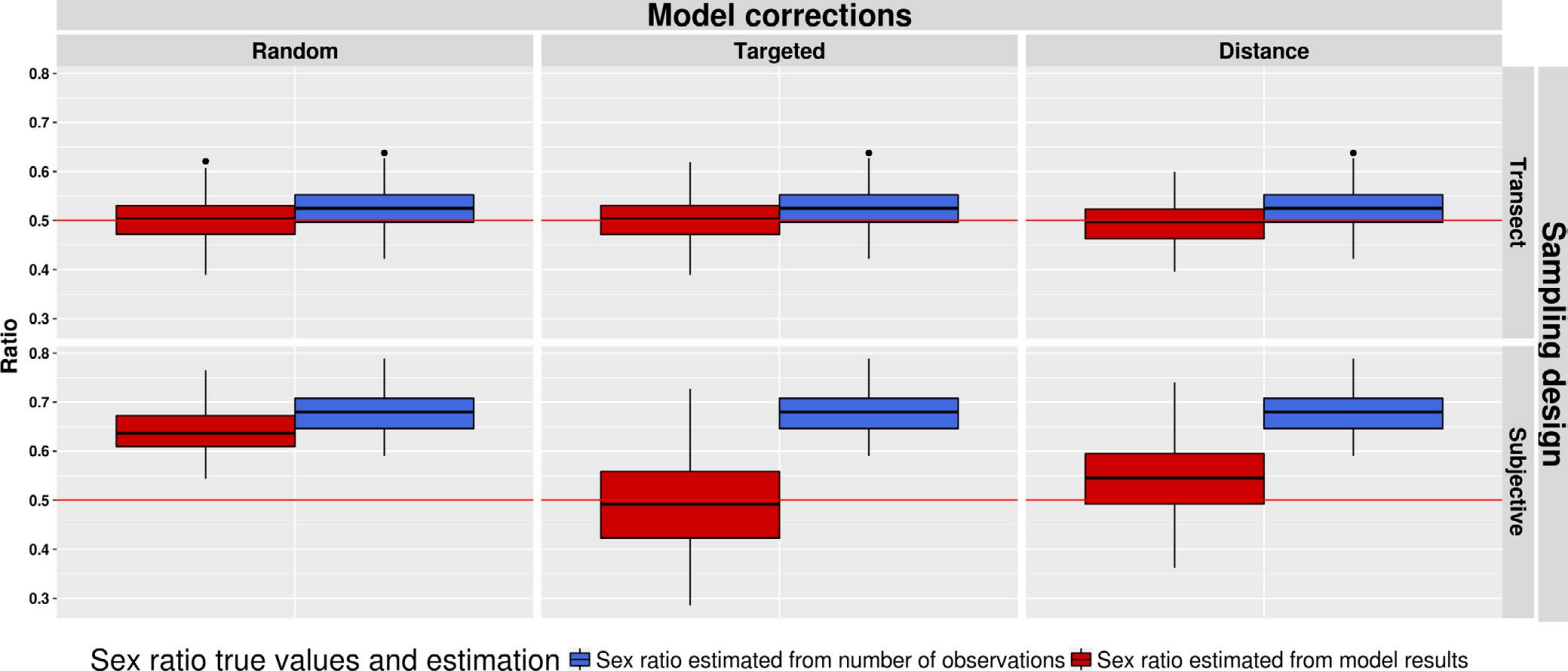
Sex ratio median and quartiles ranges estimated for the two sampling designs (systematic and subjective) in the virtual case study from the number of observations (blue) and from predictions of SDMs (red) without (random background point) and with spatial bias correction methods (targeted background point and distance to trajectories). The red line represents the mean sex ratio calculated from all simulated presences (true value)

For the systematic sampling design, the estimated sex ratios, based on either raw observations or un‐corrected SDMs, were already very close to the true value, but the use of any correction method improved this estimation (Figure 4). However, for the subjective design, the sex ratio was highly biased in favor of one sex when it was estimated from the raw number of observations or from the expected total number of signs predicted from a noncorrected model. Both correction methods improved the estimation of sex ratio, the targeted background method resulting in the least biased estimates. As for the estimation of the model parameters, the variance of the ratio estimation increased when a correction method was applied.

Sex ratio estimation depends on the estimated detection probabilities. We found that the distance to trajectories method estimated lower distance decay of observation probability than the targeted background point method (Figures A5.1 and A5.2).

#### 3.1.4 Predicted maps

Female predicted relative intensity maps were in all cases highly correlated with the true simulated distribution (Spearman correlation coefficient *ρ* > 0.94; Table 3). For male‐predicted maps, correlations were also high (*ρ* > 0.90) except in the case of subjective sampling without corrections (*ρ* = 0.37) highlighting the effect of corrections on male models. The predicted maps created from generic models were compared to female and male distributions. Generic predicted maps were poorly correlated to male distribution in all cases (*ρ* < 0.21) with higher values observed for the random and systematic sampling design. However, the generic‐predicted maps showed higher correlation with the female‐simulated distribution with higher values for the subjective design (*ρ* 0.33–0.71).

**Table 3:** Spearman correlation coefficient calculated between predicted maps and simulated distribution for sex‐specific and generic models

Design	Correction	Comparison (predicted/simulated)	Mean Spearman correlation	Standard deviation of Spearman correlation
Random	Random	Female/Female	0.99	0.01
Systematic	Random		0.98	0.01
	Targeted		0.99	0.01
	Distance		0.99	0.01
Subjective	Random		0.94	0.02
	Targeted		0.95	0.05
	Distance		0.95	0.04
Random	Random	Male/Male	0.99	0.01
Systematic	Random		0.99	0.01
	Targeted		0.99	0.01
	Distance		0.99	0.01
Subjective	Random		0.38	0.10
	Targeted		0.93	0.09
	Distance		0.91	0.09
Random	Random	Generic/Female	0.33	0.35
Systematic	Random		0.66	0.28
	Targeted		0.56	0.38
	Distance		0.66	0.39
Subjective	Random		0.85	0.04
	Targeted		0.70	0.10
	Distance		0.71	0.11
Random	Random	Generic/Male	0.21	0.36
Systematic	Random		−0.23	0.36
	Targeted		−0.11	0.46
	Distance		−0.26	0.48
Subjective	Random		−0.40	0.08
	Targeted		−0.28	0.15
	Distance		−0.33	0.17

### 3.2 Capercaillie case study

Capercaillie models had mostly poor performances, ranging from AUC = 0.43 to AUC = 0.59. These AUC values are very low (a value of 0.5 corresponds to random predictions), but are largely due to the fact that we restricted background sampling around the surveyed areas. These were located preferentially in suitable habitat, and thus differentiating degrees of habitat quality are difficult. AUC values where higher when comparing observed signs with uniform random background points (AUC = 0.69 to AUC = 0.86). Male models performed overall better than female models in the case of subjective design but performed worse in the case of systematic design (Table S3.2). The lowest performance was observed for the subjective design with distance to trajectories correction male model (AUC = 0.43). For males, the subjective design with targeted corrections performed best (AUC = 0.54), and for females, the systematic design without correction had the best performance (AUC = 0.59). Generic models showed higher performance than the male model with systematic design and had overall similar performances compared to female models.

The sex ratio was closer to a value of 0.5 in the case of systematic sampling, using either the number of signs, the noncorrected model (systematic design), or the targeted background point correction (both designs; Table 4). However, the estimated sex ratio obtained with the distance to trajectories method was lower for both systematic and subjective design (0.39 and 0.34, respectively).

**Table 4:** Sex ratio (proportion of females) estimates for Capercaillie

	Number of observations	Model, noncorrected	Model, targeted background	Model, distance to trajectories
Systematic	0.41	0.42	0.41	0.39
Subjective	0.33	0.34	0.41	0.34

**Figure 5:**
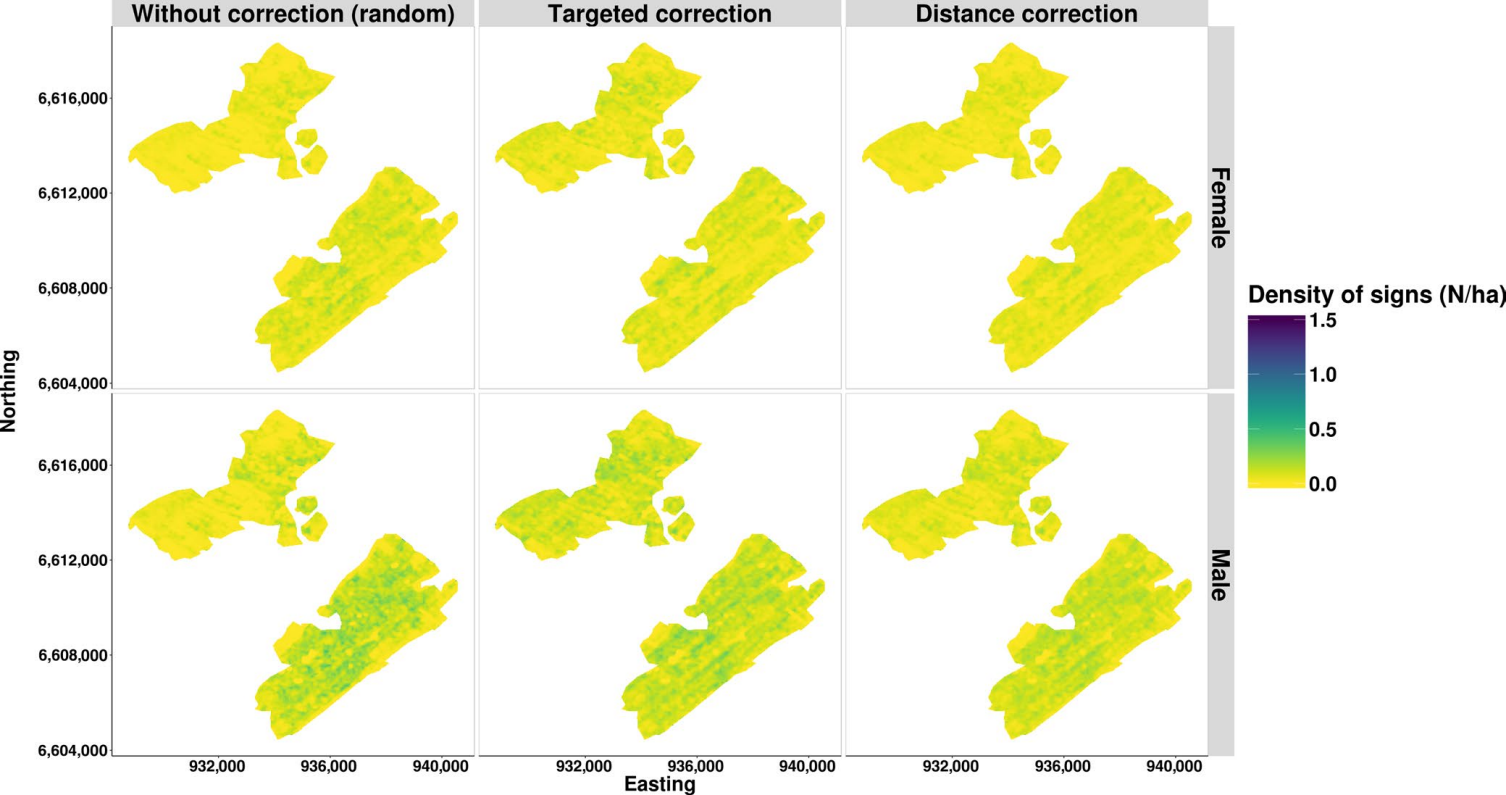
Capercaillie predicted density of signs (N/ha) for both sexes in the case of subjective sampling

Maps of the predicted density of signs were calculated for each model (Figure 5). Maps of the generic models were overall highly correlated to both female and male predicted maps (Spearman correlation coefficient *ρ* > 0.81; Table 5). Models from systematic sampling design had lower correlation than models from subjective sampling. Generally, subjective models' predictions were better correlated to simulated densities for females than for males. The generic model maps were also compared to the sum of male and female density maps (Figure A4.1). Correlations between the predictions were high (*ρ* > 0.92), with higher values for the three subjective cases (*ρ* > 0.99). Furthermore, the differences between the two maps were overall low with the root–mean–squared error lower than 0.016 signs/ha for the case of targeted background sampling (Figure A4.2).

**Table 5:** Spearman correlation coefficient calculated between predicted maps of sex‐specific model and prediction from generic models for Capercaillie

Design	Sex	Correction	Mean Spearman correlation
Systematic	Female	Random	0.81
		Targeted	0.81
		Distance	0.76
Systematic	Male	Random	0.95
		Targeted	0.92
		Distance	0.93
Subjective	Female	Random	0.98
		Targeted	0.97
		Distance	0.97
Subjective	Male	Random	0.98
		Targeted	0.91
		Distance	0.95

## 4 DISCUSSION

The aim of this study was to evaluate the effect of two different spatial bias correction methods on species sex ratio estimations (and more generally spatially segregated subgroups), SDM parameter estimation and prediction accuracy. The correction methods were tested on two sampling designs: a transect and a subjective sampling with two datasets: a virtual species and a sexually dimorphic species (Capercaillie).

The systematic sampling design allowed a better estimation of the parameters with or without corrections for potential spatial bias, as it covers a larger part of the environmental variation in the landscape and forces the observer to also visit areas with expected lower habitat quality, thus reducing possible spatial bias (Cardador et al., 2017). The use of real observer trajectories to sample a virtual species confirmed that a non‐negligible sampling bias can occur in the absence of a predefined sampling design, in coherence with our hypothesis H1. The knowledge of observer trajectories during their survey associated with each method of bias correction allowed a better estimation of model parameters in all cases, in line with our hypothesis H3. Those results are highlighting the importance of having a good knowledge of the sampling effort to produce more accurate models (Kramer‐Schadt et al., 2013; Ranc et al., 2017). However, those better estimations are made at the cost of an increase of the variance of the parameter estimations. The well‐known targeted background point method performed overall better than the alternative method using “distance to trajectories” as predictor variable combined with random background points. However, differences between these methods might be more pronounced in other configurations, as the overall effect of bias correction also depends on the modeling technique (Thibaud, Petitpierre, Broennimann, Davison, & Guisan, 2014), on the range width (Ranc et al., 2017), and on the sample size (Thibaud et al., 2014).

The estimation of the sex ratio using a virtual species showed that a major bias can occur when the two sexes differ in their habitat preferences, supporting our hypothesis H2. The use of systematic sampling allowed a correct estimation of the ratio, which was even better when correction methods were applied. However, when the observations were sampled using a subjective design, the ratio was highly biased toward one sex. The application of the two correction methods allowed a more reliable but not fully corrected estimation of this ratio, as was observed by Syfert, Smith, and Coomes (2013). The targeted background correction method gave better ratio estimates in the virtual case study. This may be due to a better ability to represent the spatial observation bias. The distance to trajectories method yielded fewer extreme predictions of spatial observer bias as it more closely followed the simulated decay of detectability with distance from sampling locations (Figures A5.1 and A5.2).

The effect of sampling bias on model accuracy was also confirmed by the finding that generic models (combining female and male observations) generated distributions highly correlated with the virtual distribution of females but not males, thus yielding biased predictions (H4). The effect of the corrections was also observed on the prediction accuracy as it was higher when corrections were applied, in particular in the case of male models.

As expected, our results indicate that if the difference between sexes is not taken into account when the dataset is spatially biased, it is likely that the full range of the potential habitat will not be covered. This can greatly impact future conservation actions (such as creation of protected areas, habitat restauration) by lowering the importance of one type of habitat or the impact of anthropogenic activities on a part of a population (Conde et al., 2010; Jiménez et al., 2017). Still, if models are developed for each sex separately with a correction for spatial sampling bias, the resulting prediction can be accurate, as demonstrated with the high correlation between the sex‐specific‐predicted map to the simulated distributions. To conclude, these results using a virtual species show that if the implementation of a systematic design is not possible, the application of a correction method such as the two tested in this study is a viable option.

In our case study on Capercaillie, the sex ratio was leaning toward males for both sampling designs. This ratio was closer to the value for a balanced sex ratio for the observations collected with the systematic design, in line with our hypothesis H5. Proportionally, more female signs were collected with this protocol than with the subjective one. However, the number of observations available for the systematic design was very low (29), thus more data are needed to confirm this pattern. Nevertheless, the ratio of males observed with systematic sampling (0.59) was slightly lower than the one reported by previous studies (0.63 and 0.625) that used subjective sampling (Mollet et al., 2015; Morán‐Luis et al., 2014). Only the targeted correction methods in the case of subjective sampling had the expected effect on the estimated sex ratio, whereas the other cases showed lower value than observed from the number of observations. Thus, our hypothesis H6 was not supported by our results, but it underlines the potential differences in efficiency of the two tested correction methods. The targeted background point method again produced a stronger decay of observation probability with distance from sampling transect than the distance to trajectories models (Figure A5.3). We note that this difference is not inherent in the two methods, but a modeling choice, it would be possible to use a simpler distance‐decay distribution in the targeted background point method or a more complex distance‐decay function in the distance to trajectories models.

The lack of efficiency of the correction method using Capercaillie observations may also be due to other factors, such as the limited number of observations or the pertinence of the environmental variables used in the model (Johnson & Gillingham, 2005), which can influence the overall model accuracy. Indeed, with the Capercaillie models important elements such as the tree species or human disturbances are not taken into account (Coppes, Judith, Dominik, Rudi, & Veronika, 2017; Sachot, Perrin, & Neet, 2003), contrary to the virtual species case where all the variables influencing the distribution were known. In addition, even if Maxent is known to be robust to sample size effects (Hernandez, Graham, Master, & Albert, 2006), a datasets with fewer than 30 observations can give inconsistent prediction (Wisz et al., 2008). The production of overall inaccurate models may limit the effects of the correction methods, and indeed, some observed model performance were poor (AUC < 0.5). We can also assume that a lower than expected difference in habitat use between sexes is occurring in Capercaillie populations, as we observed high correlation between sex‐specific and generic models and low difference between generic and the sum of sex‐specific predictions maps, in contrast to our hypothesis H7. In addition, though the same forest areas were surveyed, some parts of the forest were covered only with systematic or only with subjective design (Figure 1), limiting the direct comparability of the estimated sex ratios.

Even if the differences in habitat preferences between sexes for Capercaillie were small in this study, many other species show more differentiated habitat use, which could affect model accuracy more strongly. This was observed in mark‐recapture studies (McKnight & Ligon, 2017) and telemetry studies (Kolts & McRae, 2017). While systematic protocols are more robust to differences between sexes, their use is unlikely to solve the entire problem of biased detectability. Despite the spatial sampling bias, differences in detection probability can still introduce a bias toward one sex in models (Guillera‐Arroita, 2016; Lahoz‐Monfort, Guillera‐Arroita, & Wintle, 2014). This limitation is well known in studies that aim to estimate population size (McKnight & Ligon, 2017). In the specific case of Capercaillie, differences in behavior are related to the cryptic plumage associated with a smaller size of female leading to a difference in predation avoidance strategy between sexes. Female presence in denser vegetation cover may induce a lower detection rate in such environments, combined with a tendency to avoid long movements and a therefore more restricted spatial distribution of droppings that further reduce the chance of observing signs (Mollet et al., 2015). Furthermore, it has been observed that females have a gregarious behavior in winter, leading to the joint presence of up to three individuals in a single roosting tree, which cannot be reliably detected by the observation of signs only such as dropping.

## 5 CONCLUSION

The use of subjective sampling protocols can yield biased SDM results, when working with species where sexes have different habitat use behavior. When using a subjective sampling design, the habitat preferred by one sex can be under‐represented in the dataset, introducing bias in the predictions from SDMs. The two spatial bias correction methods tested here were able to correct this effect in a virtual case study, but not in the case of a forest bird, Capercaillie. The effectiveness of spatial correction methods cannot be taken for granted and other potential sources of bias, such as sex differences in detectability, require additional attention. We argue that spatial correction methods can nevertheless be useful when developing SDMs for subgroups (e.g., sex and age) with distinct behaviors but that the accuracy of the prediction depends on their ability to well represent the spatial observation bias.

## CONFLICT OF INTEREST

The authors declare no conflict of interest.

## AUTHORS' CONTRIBUTIONS

A.G. co‐designed the methodology, analyzed the data, and wrote the manuscript. J.‐M. M., J.P., and B.R. designed the methodology and contributed to model development and data analyses. J.‐M. M. developed the original code for LiDAR variable extraction. All authors contributed to and gave approval for the final manuscript.

## Data Availability

Data and R code are available at the repository: (https://gitlab.irstea.fr/anouk.glad/Spatial_sampling_bias). The Capercaillie observation dataset is available on request only due to the conservation status of this species in the Jura Massif in France. Please send your request to Ms Depraz at groupe-tetras@wanadoo.fr for this dataset. R code to calculate LiDAR metrics is available at (https://gitlab.irstea.fr/jean-matthieu.monnet/lidaRtRee).
