# Supplementary material for: Importance and effectiveness of correction methods for spatial sampling bias in species with sex‐specific habitat preference
Source: Ecol Evol. 2019 Nov 19;9(23):13188–201. doi: 10.1002/ece3.5765 (PMC6912901; doi:10.1002/ece3.5765)

**Appendix**

**Appendix S1**

Figure S1.1: Density curves for each environmental variable and each sampling design, showing the differences in coverage of the study area for the virtual species: overall landscape (red), systematic transect (blue), and subjective trajectories (green).


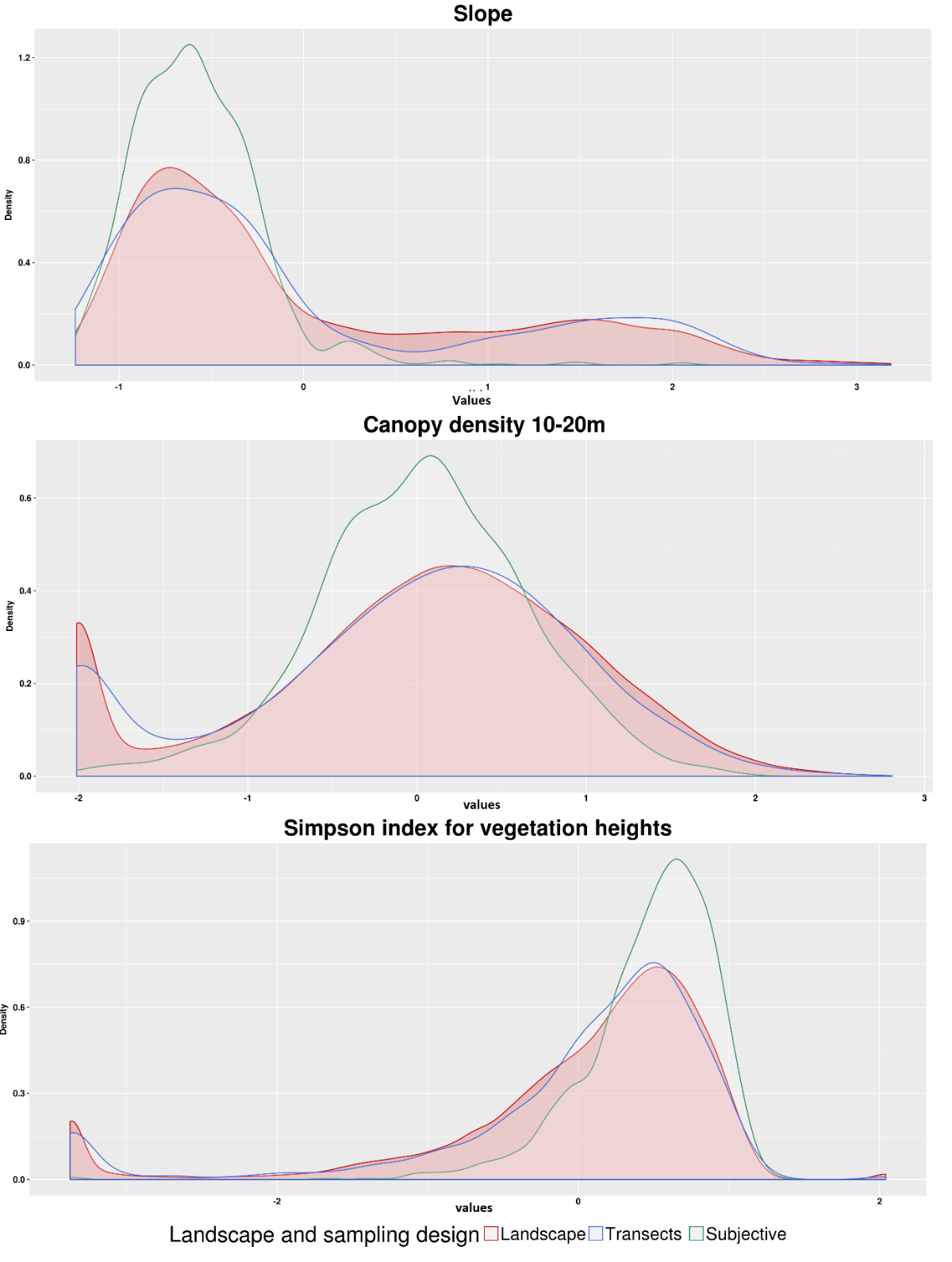


**Appendix S2**

Fig. S2.1: Parameter estimates (100 replicate models) for virtual sub-group 2 (male). The red line represents the true parameter value.


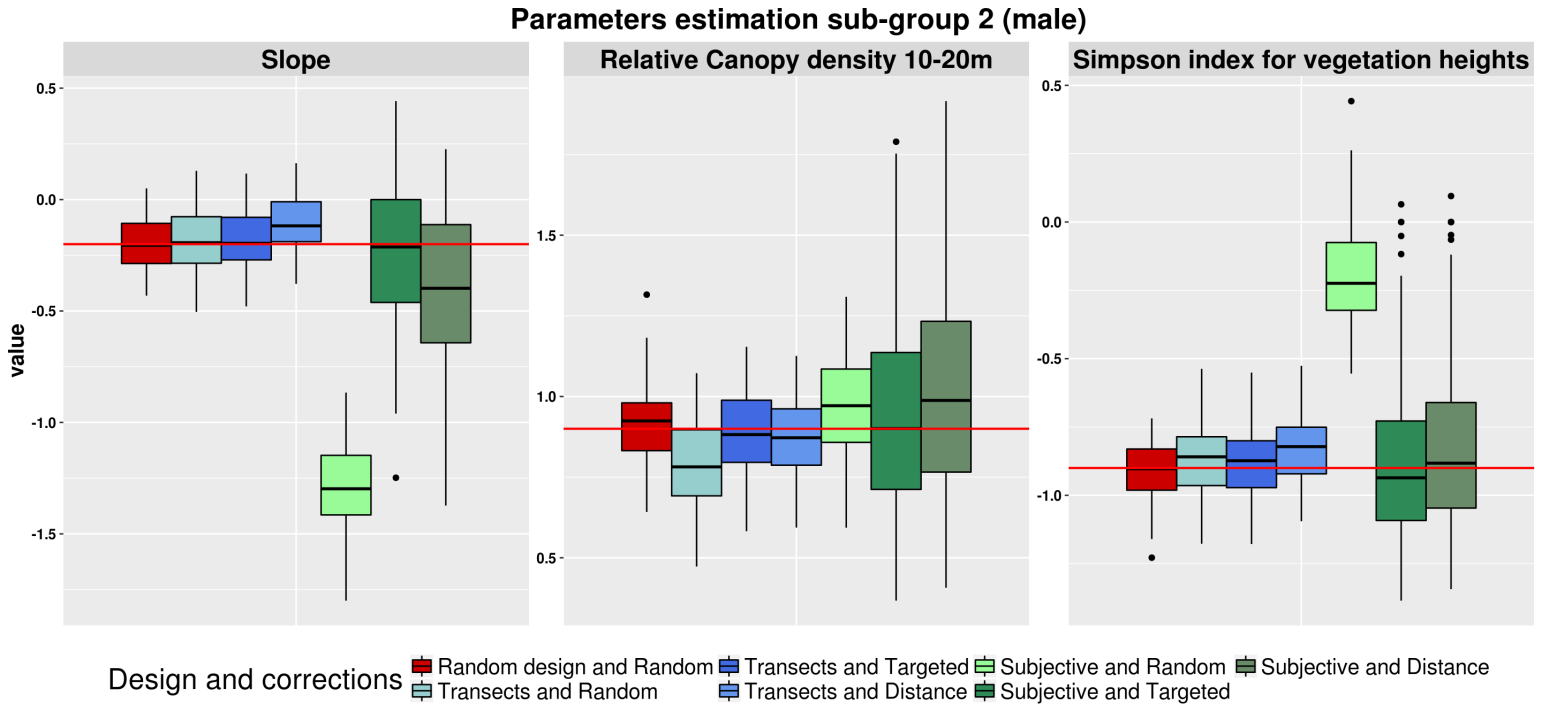


**Appendix S3**

Table S3.1: AUC values for all virtual species models (sex-specific and generic).

| Sex | Design | Correction | AUC | Standard error |
| --- | --- | --- | --- | --- |
| Female | Random | Random | 0.82 | 0.02 |
|  | Systematic | Random | 0.83 | 0.02 |
|  |  | Targeted | 0.83 | 0.02 |
|  |  | Distance | 0.80 | 0.02 |
|  | Subjective | Random | 0.57 | 0.03 |
|  |  | Targeted | 0.58 | 0.03 |
|  |  | Distance | 0.56 | 0.03 |
| Male | Random | Random | 0.78 | 0.02 |
|  | Systematic | Random | 0.78 | 0.02 |
|  |  | Targeted | 0.78 | 0.02 |
|  |  | Distance | 0.75 | 0.03 |
|  | Subjective | Random | 0.55 | 0.04 |
|  |  | Targeted | 0.57 | 0.04 |
|  |  | Distance | 0.56 | 0.04 |
| Generic | Random | Random | 0.56 | 0.02 |
|  | Systematic | Random | 0.58 | 0.02 |
|  |  | Targeted | 0.58 | 0.02 |
|  |  | Distance | 0.56 | 0.02 |
|  | Subjective | Random | 0.54 | 0.02 |
|  |  | Targeted | 0.56 | 0.02 |
|  |  | Distance | 0.55 | 0.02 |

Table S3.2 AUC values for all Capercaillie models (sex-specific and generic).

| Sex | Design | Correction | AUC | Standard error |
| --- | --- | --- | --- | --- |
| Female | Systematic | Random | 0.59 | 0.05 |
|  |  | Targeted | 0.56 | 0.02 |
|  |  | Distance | 0.44 | 0.04 |
|  | Subjective | Random | 0.48 | 0.03 |
|  |  | Targeted | 0.53 | 0.03 |
|  |  | Distance | 0.57 | 0.03 |
| Male | Systematic | Random | 0.45 | 0.04 |
|  |  | Targeted | 0.46 | 0.04 |
|  |  | Distance | 0.43 | 0.06 |
|  | Subjective | Random | 0.52 | 0.02 |
|  |  | Targeted | 0.54 | 0.02 |
|  |  | Distance | 0.50 | 0.02 |
| Generic | Systematic | Random | 0.57 | 0.04 |
|  |  | Targeted | 0.59 | 0.04 |
|  |  | Distance | 0.53 | 0.05 |
|  | Subjective | Random | 0.50 | 0.01 |
|  |  | Targeted | 0.51 | 0.01 |
|  |  | Distance | 0.52 | 0.06 |

Appendix S

Fig. S4.1 Predicted density of Capercaillie signs for the subjective design with targeted background point correction: a) genericmodel, b) sum of sign density from the two sex-specific models (see Fig 5 for sex-specific maps).

**
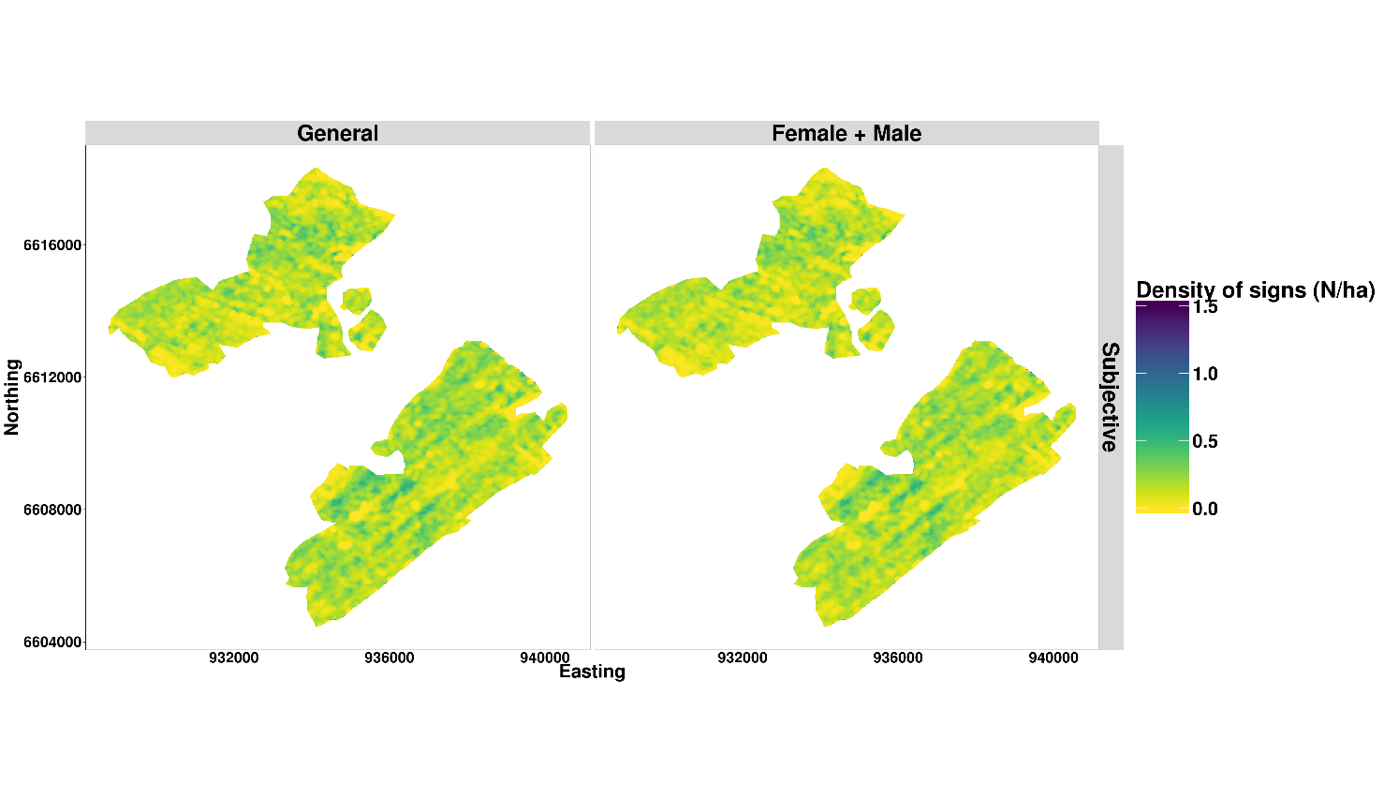
**

Fig S4.2: Difference in predicted Capercaillie sign density between generic model and the sum of the two sex-specific models.


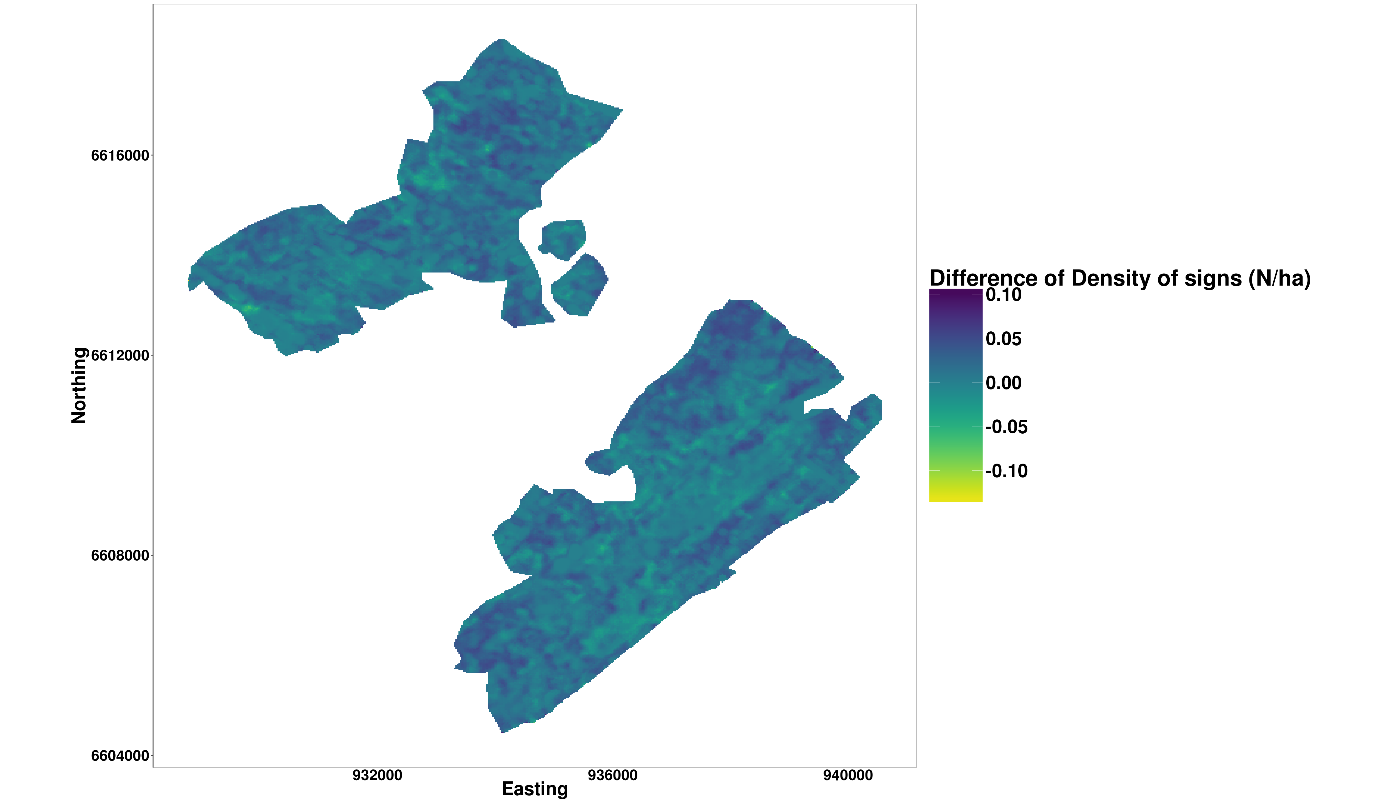


Appendix S5

Fig. S5.1 Sign detection probability as a function of distance to trajectories: logspline density estimation underlying target-background point method (red), and estimated by the model with distance to trajectories corrections (blue), for virtual species female with systematic sampling.


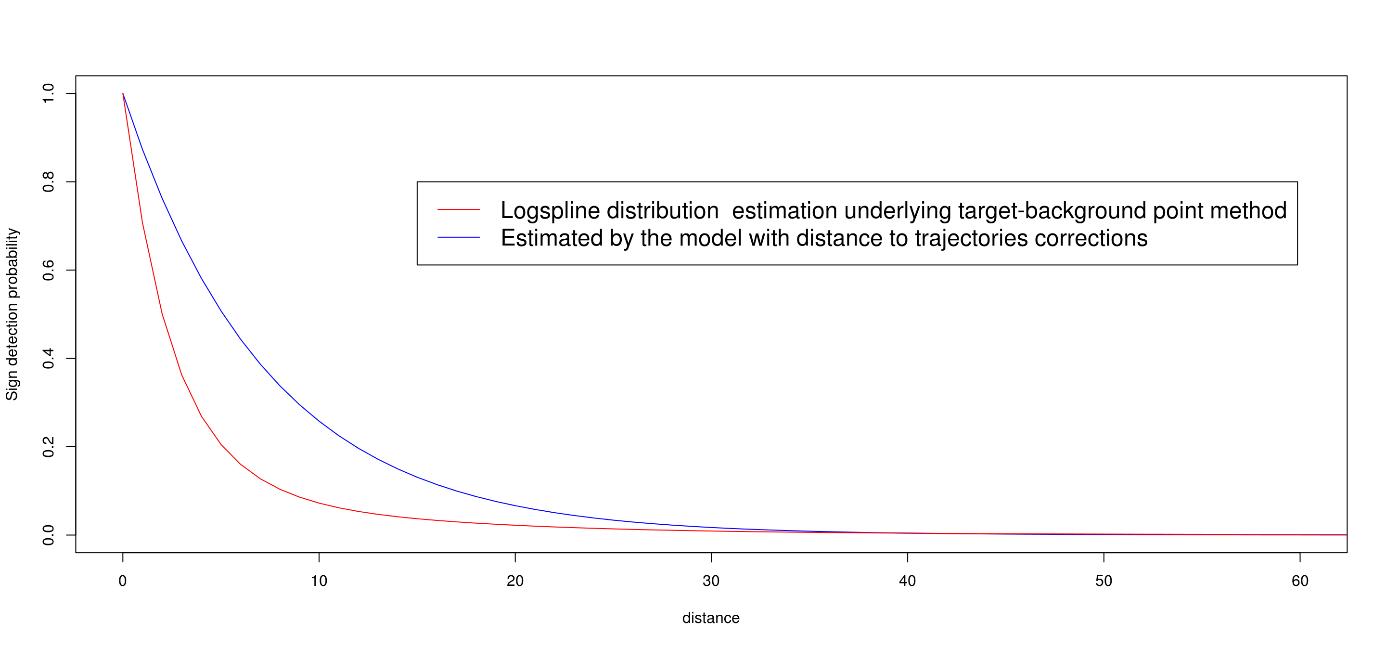


Fig. S5.2: Sign detection probability as a function of distance to trajectories: logspline density estimation underlying target-background point method (red), and estimated by the model with distance to trajectories corrections (blue), for virtual species female with subjective sampling.


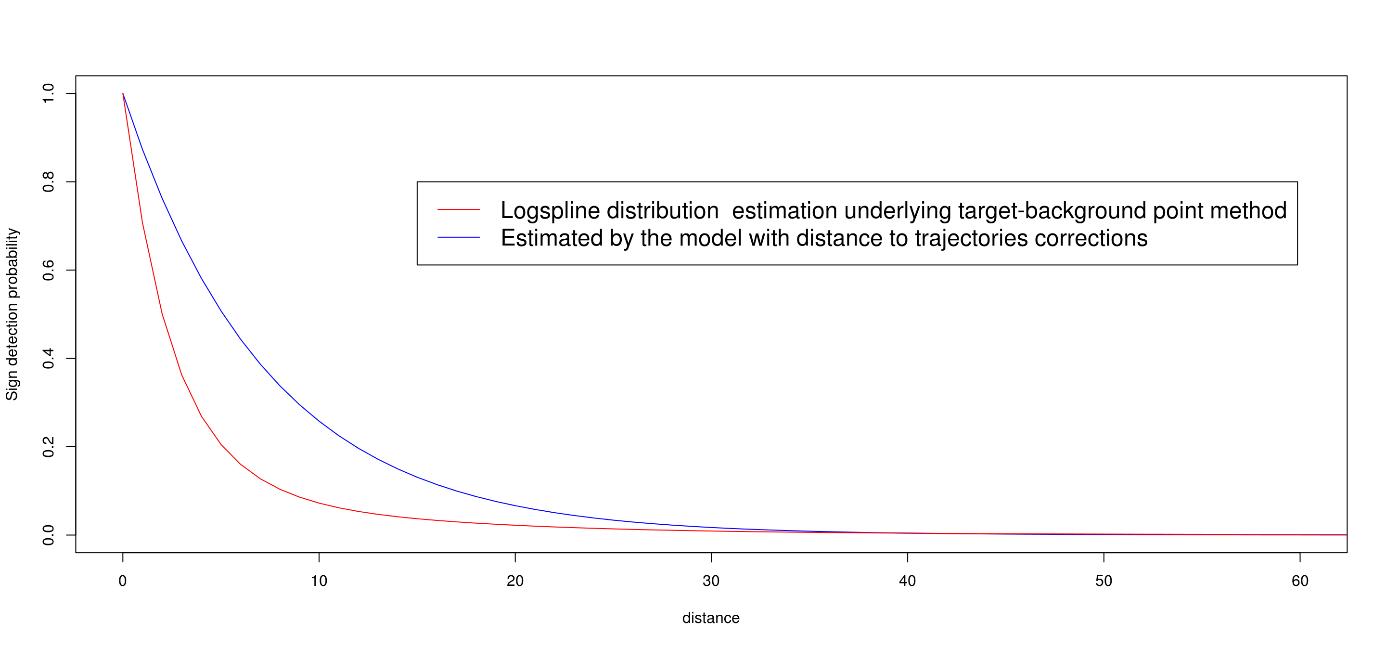


Fig. S5.3: Sign detection probability as a function of distance to trajectories: logspline density estimation underlying target-background point method (red) and estimated by distance corrected model for the two samplings design of Capercaillie and the different type of model (female, male, generic).


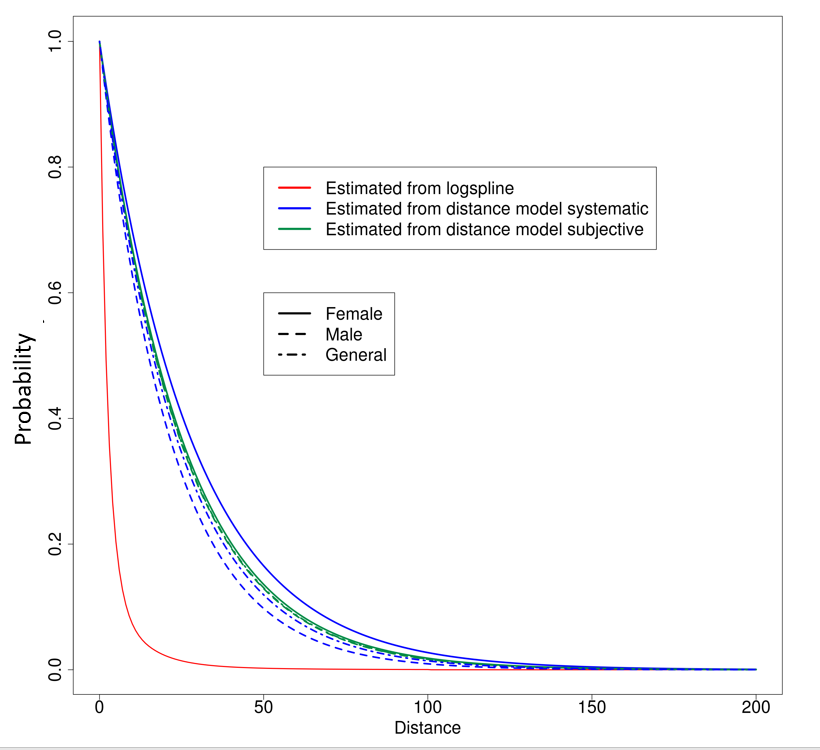

Supplement: Supplementary file 1 [file ECE3-9-13188-s001.docx]
